# A method to improve binary forecast skill verification

**DOI:** 10.1016/j.mex.2024.103010

**Published:** 2024-10-15

**Authors:** Thitithep Sitthiyot, Kanyarat Holasut

**Affiliations:** a Department of Banking and Finance, Faculty of Commerce and Accountancy, Chulalongkorn University, Mahitaladhibesra Bld., 10th Fl., Phayathai Rd., Pathumwan, Bangkok 10330, Thailand; b Department of Chemical Engineering, Faculty of Engineering, Khon Kaen University, Mittapap Rd., Muang District, Khon Kaen 40002, Thailand

**Keywords:** Binary event, Deterministic forecast evaluation, Direction-of-change, Skill score, A method to improve binary forecast skill verification

## Abstract

To overcome the limitations of existing deterministic binary forecast skill verification methods that award a perfect score for forecasting events considered easy to forecast, an improvement factor is introduced. It comprises two components which are 1) a measure of the ease with which an event can be accurately forecasted and 2) a measure of frequency of event. By using two hypothetical datasets, this study demonstrates that an improvement factor could enhance the performance of existing deterministic binary forecast skill verification methods by awarding score that is close to score for no-skill forecast for the perfect forecasts of events considered easy to forecast. In addition, the forecast and actual data on annual inflation rate are used to demonstrate how an improvement factor could be used together with the existing deterministic binary forecast skill verification methods in order to assess skills of the forecasters in practice.

• Existing deterministic binary forecast skill verification methods fail to award correct score for events considered easy to forecast.
• An improvement factor is developed in order to enhance performance of existing deterministic binary forecast skill verification methods.
• Hypothetical and empirical data are used to validate how an improvement factor works.

## Graphical abstract

**Figure:**
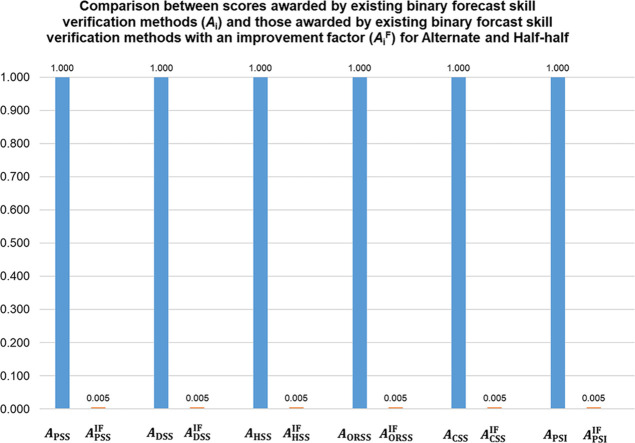

Specification table

**Table:** 

Subject area:	Economics and finance
More specific subject area:	Directional forecast verification
Name of method:	A method to improve binary forecast skill verification
Name and reference of original method:	Not applicable
Resource availability:	All data analyzed in this study are available in Supplementary material.

## Background

Forecast is important for scientific, economic, and administrative purposes [1] since reliable forecast could help to reduce uncertainty about the future [2]. To evaluate skill in deterministic binary forecast, a tool widely used in numerous scientific disciplines is deterministic forecast skill verification method [3]. As discussed in Sitthiyot and Holasut [3], examples include computer science – the performance of hybrid decision forest in short-term forecasting [4]; ecology – the measurement of the degree to which two different species are associated in nature [5]; economics – the forecasting of binary outcomes [6]; environment – the forecast of visibility degradation due to poor air quality [7]; finance – the prediction of stock market returns [8]; hydrology – the development and verification of a real-time stochastic precipitation nowcasting system for urban hydrology [9]; machine learning – the development and application of spatiotemporal machine learning/data mining techniques [10]; the study of machine learning in space weather [11]; medical and clinical studies – the mammogram screening [12]; the study of predictive models for decision-making against dengue haemorrhagic fever epidemics [13]; meteorology – the behavior of verification measures with respect to random changes [14]; seismology – earthquake forecasting and verification [15]; and space weather – the study of an operational solar flare forecast [16].

According to Jolliffe and Stephenson [17], a number of deterministic binary forecast skill verification methods have been continually invented and/or reinvented over the past century. However, Wilks [18] and Murphy [19] note that deterministic binary forecast skill verification methods frequently used are Peirce skill score (PSS) [20], Dolittle skill score (DSS) [21], Heidke skill score (HSS) [22], Odds ratio skill scores (ORSS) [23], and Clayton skill score (CSS) [24]. Sitthiyot and Holasut [3] recently introduced another deterministic binary forecast skill verification method called Prediction Skill Index (PSI) which has been applied by Sitthiyot and Holasut [25] in order to evaluate skills in forecasting direction-of-changes of growth of real gross domestic product and inflation rate simultaneously.

While the existing deterministic binary forecast skill verification methods both conventional and recently introduced ones can be used to assess skills of forecasters in forecasting binary events in numerous types of events as demonstrated in Sitthiyot and Holasut [3], to our knowledge, there are two broad types of binary events considered somewhat easy to forecast and the forecasters should be awarded scores that are close to or no different from score for no-skill forecast. However, these existing deterministic binary forecast skill verification methods award a perfect score. The first type of event is the event that has constantly alternate binary outcomes, for example, a pattern of day and night cycle. The second type of event is the event where approximately the first half of binary outcomes belongs to one category and roughly the second half of binary outcomes belongs to the other category. An example of this type of event is daily average temperature in a certain area. For example, the city of Rome in Italy has the daily average temperature that is above 14 °C from May to October while, from November to April of the following year, the daily average temperature in Rome is equal to or below 14 °C [26].

To overcome the limitations of existing deterministic forecast skill verification methods in evaluating skills in forecasting these two types of binary events considered easy to forecast as discussed above and also to fill the gap of knowledge in the field of forecast verification, an improvement factor is devised. It comprises two components which are (1) a measure of the ease with which an event can be accurately forecasted and (2) a measure of frequency of event. Given the use of deterministic binary forecast skill verification methods in various scientific disciplines as discussed above, an improvement factor could be useful for forecasters who develop models and/or machine learning algorithms in order to forecast binary events. In addition, a measure of the ease with which an event can be accurately forecasted can be used as a standalone method to preliminarily check whether the nature of event is easy or difficult to forecast accurately.

## Method details

For notations, let a,b,c,anddbe the cell counts for *Hits, False alarms, Misses*, and *Correct rejections*, respectively. In addition, let n be the total number of observations. Table 1 shows the 2 × 2 contingency table of forecasted/observed outcomes of any given event that are used to calculate a score awarded by a particular deterministic binary forecast skill verification method.

**Table 1:** The 2 × 2 contingency table of forecasted/observed outcomes.

*Forecasted outcomes*	*Observed outcomes*		Total
	*Yes*	*No*	
***Yes***	a	b	a+b
	*(Hits)*	*(False alarms)*	
***No***	c	d	c+d
	*(Misses)*	*(Correct rejections)*	
**Total**	a+c	b+d	n=a+b+c+d

This study chooses PSS, DSS, HSS, ORSS, CSS, and PSI in order to demonstrate that, despite PSS, DSS, HSS, ORSS, CSS being frequently used as noted by Wilks [18] and Murphy [19] and PSI being recently introduced, these deterministic binary forecast skill verification methods fail to give the correct scores for forecasting two types of events considered easy to forecast which are (1) the event that has constantly alternate binary outcomes and (2) the event where the first half of binary outcomes belongs to one category and the second half of binary outcomes belongs to the other category. In addition, the scores awarded by these deterministic binary forecast skill verification methods can be conveniently compared since their range is between −1 and 1, where −1 means the poorest forecast skill and 1 means the perfect forecast skill. Table 2 provides definitions and ranges of PSS, DSS, HSS, ORSS, CSS, and PSI.

**Table 2:** Definitions and ranges of deterministic binary forecast skill verification methods.

Methods	Definitions	Ranges
Peirce skill score (PSS)	$\frac{ad-bc}{\left(b+d)(a+c\right)}$	[−1, 1]
Dolittle skill score (DSS)	$\frac{ad-bc}{\sqrt{\left(a+b)(c+d)(a+c)(b+d\right)}}$	[−1, 1]
Heidke skill score (HSS)	$\frac{a+d-\left[\frac{\left(a+b)(a+c\right)}{n}\right]-\left[\frac{\left(b+d)(c+d\right)}{n}\right]}{n-\left(\frac{\left(a+b)(a+c\right)}{n}\right)-\left[\frac{\left(b+d)(c+d\right)}{n}\right]}$	[−1, 1]
Odds ratio skill score (ORSS)	$\frac{ad-bc}{ad+bc}$	[−1, 1]
Clayton skill score (CSS)	$\frac{a}{a+b}-\frac{c}{c+d}$	[−1, 1]
Prediction skill index (PSI)	$\frac{\left(\frac{\frac{a}{n}\;-\;\left[\left(\frac{a+b}{n}\right)\left(\frac{a+c}{n}\right)\right]}{\sqrt{\left(\frac{a+b}{n}\right)\left(\frac{a+c}{n}\right)}}\;+\;\frac{\frac{d\;}{n}-\;\left[\left(\frac{b+d}{n}\right)\left(\frac{c+d}{n}\right)\right]}{\sqrt{\left(\frac{b+d}{n}\right)\left(\frac{c+d}{n}\right)}}\right)\;-\;\left(\frac{\frac{b}{n}\;-\;\left[\left(\frac{a+b}{n}\right)\left(\frac{b+d}{n}\right)\right]}{\sqrt{\left(\frac{a+b}{n}\right)\left(\frac{b+d}{n}\right)}}\;+\;\frac{\frac{c}{n}\;-\;\left[\left(\frac{a+c}{n}\right)\left(\frac{c+d}{n}\right)\right]}{\sqrt{\left(\frac{a+c}{n}\right)\left(\frac{c+d}{n}\right)}}\right)}{2}$	[−1, 1]

According to Sitthiyot and Holasut [25], forecast skill is typically interpreted as the percentage improvement over the reference forecast and presented as a skill score, not in the form of statistical significance. Let $SS_{i}$ denote skill score for a particular deterministic binary forecast verification method i, $A_{i}$ denote the score awarded by a particular deterministic binary forecast verification method i, $A_{i}^{\text{perf}}$ denote the score awarded by a particular deterministic binary forecast verification method i that could be achieved by the perfect forecast. In addition, let $A_{i}^{\text{ref}}$ denote the score for the reference forecast. In this study, the random forecast is used as a reference forecast whose value is set to be 0. Note, however, that other benchmarks such as always forecasting *Up, Down*, or *No-change* can also be used as a baseline forecast since PSS, DSS, HSS, ORSS, CSS, and PSI also award these persistence forecasts the value of 0 in all three cases as demonstrated in Sitthiyot and Holasut [3]. According to Wilks [18], in generic form, skill score characterized by a particular deterministic binary forecast skill verification method $\left(SS_{i}\right)$, with respect to the reference forecast $\left(A_{i}^{\text{ref}}\right)$, can be calculated as shown in Eq. (1).

(1) $$SS_{i}\;=\left(\frac{A_{i}-\;A_{i}^{\text{ref}}}{A_{i}^{\text{perf}}-\;A_{i}^{\text{ref}}}\right)*100\%$$

If $A_{i}$ equals $A_{i}^{\text{perf}}$, the skill score for a particular deterministic binary forecast skill verification method i attains its maximum value of 100 %. If $A_{i}$ equals $A_{i}^{\text{ref}}$, the skill score for a particular deterministic binary forecast skill verification method i is equal to 0 %, suggesting that there is no improvement over the reference forecast. If the forecasted outcome based on a particular deterministic binary forecast skill verification method i being evaluated is inferior to the reference forecast, its skill score is <0 %. Provided that $A_{i}^{\text{perf}}$ and $A_{i}^{\text{ref}}$ for a particular deterministic binary forecast skill verification method i used in this study are set to be equal to 1 and 0, respectively, $SS_{i}$ can be computed as shown in Eq. (2).

(2) $$SS_{i}=A_{i}*100\%.$$

To overcome the limitations of existing deterministic binary forecast skill verification methods in that they award a perfect score for the forecasts of events that are considered easy to forecast, an improvement factor (IF) is introduced. IF has two components which are 1) a measure of the ease with which an event can be accurately forecasted (EEF) and 2) a measure of frequency of event (FE). The definitions of and the justifications for EEF and FE as well as the way in which IF is derived are as follows.

### A measure of the ease with which an event can be accurately forecasted

Let AS denote the accuracy score which is computed as shown in Eq. (3).

(3) $$\begin{array}{ccc} \text{AS} & = & \frac{Hits\;\left(a\right)\;+\;Correct\;rejections\;\left(d\right)}{Total\;number\;of\;observations\;\left(n\right)}\\ & & 0\leq \text{AS}\leq 1 \end{array}$$

The value of AS lies between 0 and 1. The closer the AS is to 0, the less accurate the forecast while the closer the AS is to 1, the more accurate the forecast. Given the definition of AS as specified in Eq. (3), a measure of the ease with which an event can be accurately forecasted (EEF) is defined as the absolute value of the difference between accuracy scores (AS) awarded to two simple forecast methods called simple forecast method 1 (SFM1) and simple forecast method 2 (SFM2) whose forecast strategies are always opposite to each other as shown in Eq. (4).

(4) $$\begin{array}{ccc} \text{EEF} & = & \left|AS_{\text{SFM}1}-AS_{\text{SFM}2}\right|\\ & & 0\leq \text{EEF}\leq 1 \end{array}$$

The value of EEF lies between 0 and 1. The closer the EEF is to 0, the more difficult the event being forecasted whereas the closer the EEF is to 1, the less difficult the event being forecasted. The justification for EEF is that it reflects the nature of event whether it is easy or difficult to forecast accurately. If the event is easy to forecast, the value of EEF would be close to 1. If the event is difficult to forecast, the value of EEF would be close to 0. Table 3 illustrates the forecast strategies of SFM1 and SFM2 as well as the observed outcomes.

**Table 3:** Forecast strategies of SFM1 and SFM2 as well as the observed outcomes.

Period (*t*)	1	2	3	4	5	6	7	8	9	10	…
***Observed outcomes***	*Yes*	*Yes*	*Yes*	*Yes*	*No*	*No*	*No*	*Yes*	*Yes*	*Yes*	…
***Forecasted outcomes* made by SFM1**	*Yes*	*Yes*	*Yes*	*Yes*	*Yes*	*No*	*No*	*No*	*Yes*	*Yes*	…
***Forecasted outcomes* made by SFM2**	*No*	*No*	*No*	*No*	*No*	*Yes*	*Yes*	*Yes*	*No*	*No*	…

For the purpose of illustration, this study assumes that SFM1 makes a correct forecast in the first period. The forecasting strategy of SFM1 is that if SFM1 makes a correct forecast in period *t*, SFM1 will continue to make the same forecast in period *t+*1. If SFM1 makes a wrong forecast in period *t*, SFM1 will change the forecast in period *t+*1. As shown in Table 3, in period 1, SFM1 forecasts *Yes* and the observed outcome is *Yes*. Therefore, SFM1 will continue to forecast *Yes* in period 2. However, in period 5, SFM1 makes a wrong forecast by forecasting *Yes* but the observed outcome is *No*. In this case, SFM1 will forecast *No* in period 6. Since the observed outcome in period 6 is *No*, SFM1 makes a correct forecast. Therefore, SFM1 will forecast *No* in period 7. For SFM2, whatever the forecasted outcome SFM1 makes in each period, SFM2’s strategy is always to forecast the opposite. The forecasted/observed outcomes of SFM1 and SFM2 such as those exemplified in Table 3 would be recorded in the 2 × 2 contingency table whether they are *Hits*(a), *False alarms* (b), *Misses* (c), and *Correct rejections* (d). Only are the cell counts for *Hits*(a) and *Correct rejections* (d) used to calculate the values of AS for SFM1 and SFM2. The values of AS awarded to SFM1 and SFM2 are then used to compute EEF as shown in Eq. (4).

### A measure of frequency of event

Let *q* be the number of events that occur during the observation period. A measure of frequency of event (FE) is defined as shown in Eq. (5).

(5) $$\begin{array}{ccc} \text{FE} & = & \frac{2*Number\;of\;events\;occurring\;during\;the\;observation\;period\;\left(q\right)}{Total\;number\;of\;observations\;\left(n\right)}\\ & & 0<\text{FE}\leq 2 \end{array}$$

The value of FE is greater than 0 but less than or equal to 2. The closer the FE is to 0, the less frequent the event whereas the closer the FE is to 2, the more frequent the event. Note that the value of 2 is used to multiply $\frac{q}{n}$ in order to make the value of FE easy to interpret in the sense that if $\frac{q}{n}\geq 0.5$, the value of FE would be greater than or equal to 1, suggesting that the event is considered more frequent. In contrast, if $\frac{q}{n}<0.5$, the value of FE would be <1, indicating that the event is considered less frequent. For an event that never occurred, the value of FE is set to be 2. While different scientific disciplines have their own criteria for considering events to be more frequent or less frequent, Sitthiyot and Holasut [25] note that being able to accurately forecast more frequent event such as sun rise would have 100 % forecast accuracy rate but it does not require exceptional skills as opposed to being able to forecast less frequent event correctly such as financial crisis would require extraordinary skills.

### An improvement factor

Given the definitions of and the justifications for EEF and FE, an improvement factor (IF) can be computed as shown in Eq. (6).

(6) $$\begin{array}{c} \text{IF}=1-\text{min}\left(\text{EEF},\;\text{FE}\right)\\ 0\leq \text{IF}\leq 1 \end{array}$$

Note that min(EEF,FE) denotes the minimum value between EEF and FE. The value of IF, which lies between 0 and 1, would then be used to multiply the score awarded by a particular deterministic binary forecast skill verification method i $\left(A_{i}\right)$ as shown in Table 2 in order to get $A_{i}$ with an improvement factor $\left(A_{i}^{\text{IF}}\right)$ as shown in Eq. (7).

(7) $$\begin{array}{ccc} A_{i}^{\text{IF}} & = & \left\{ \begin{array}{c} \mathrm{IF}*A_{i},\;\mathrm{given}\;\mathrm{FE}\geq 0.5\\ \mathrm{IF}*\left(1.5-\left|A_{i}\right|\right),\;\mathrm{given}\;0<\mathrm{FE}<0.5\;\mathrm{and}\;A_{i}\geq 0\\ -\mathrm{IF}*\left(1.5-\left|A_{i}\right|\right),\;\mathrm{given}\;0<\mathrm{FE}<0.5\;\mathrm{and}\;A_{i}<0 \end{array}\right.\\ & & -1\leq A_{i}^{\text{IF}}\leq 1 \end{array}$$

The value of $A_{i}^{\text{IF}}$ is between –1 (the poorest forecast skill) and 1 (the perfect forecast skill) with 0 being indifferent from the random forecast which is set to be the reference forecast. Note that the values of 1.5 and 0.5 as shown in Eq. (7) are scaling factors so that the value of $A_{i}^{\text{IF}}$ would lie between –1 and 1, making it convenient when comparing among scores awarded by different deterministic binary forecast skill verification methods whose ranges are normally set to be between –1 and 1. In addition, $|A_{i}|$ is the absolute value of $A_{i}$. Similar to $A_{i}$, the forecast skill based on the value of $A_{i}^{\text{IF}}$ is interpreted as the percentage improvement over the reference forecast, not in the form of statistical significance.

In order to demonstrate that the existing deterministic binary forecast skill verification methods, namely, PSS, DSS, HSS, ORSS, CSS, and PSI equipped with IF can overcome the limitations of the original PSS, DSS, HSS, ORSS, CSS, and PSI that fail to award the correct scores for forecasting events considered easy to forecast which are the event that has constantly alternate binary outcomes (Alternate) and the event where the first half of binary outcomes belongs to one category and the second half of binary outcomes belongs to the other category (Half-half), this study generates the hypothetical datasets for these two types of events (*n* = 400 for each dataset) and assumes that the forecaster makes the perfect forecasts for these two types of events for the ease of comparing scores across deterministic binary forecast skill verification methods. In addition, the data on Thailand annual inflation rate (Inf) forecasts made by the Bank of Thailand (BOT), the Fiscal Policy Office (FPO), and the National Economic and Social Development Council (NESDC) between 2003 and 2022 (*n* = 20 for each sample) as well as the actual data on Thailand inflation rate during the same period are used to demonstrate that PSS, DSS, HSS, ORSS, CSS, and PSI equipped with IF could be used to verify skill in deterministic binary forecast in practice. Note that the forecasted/observed binary outcomes for Alternate and Half-half are either *Yes* or *No* whereas those for Inf are either *Up* or *Down*. The hypothetical data on Alternate and Half-half are included in Supplementary material. The forecast and actual data on Inf are collected from *Monetary Policy Report* published by BOT [27] and are also included in Supplementary material. Table 4 provides the descriptions and sources of data on Alternate, Half-half, Inf forecasts made by BOT, NESDC, and FPO as well as actual Inf.

**Table 4:** The descriptions and sources of data on Alternate, Half-half, Inf forecasts made by BOT, FPO, and NESDC as well as actual Inf.

Data	Descriptions	*n*	Sources
**Alternate**	Hypothetical data representing an event that has constantly alternate binary outcomes (*Yes, No, Yes, No, Yes, No, Yes, No*, …). It contains 200 *Yeses* and 200 *Noes*.	400	Authors'own data
**Half-half**	Hypothetical data representing an event where the first half of binary outcomes belongs to one category (*Yes*) and the second half of binary outcomes belongs to the other category (*No*). It contains 200 *Yeses* and 200 *Noes*.	400	Authors'own data
**Inf forecast made by BOT**	Data on Thailand annual inflation rate forecast made by the BOT between 2003 and 2022.	20	BOT [27]
**Inf forecast made by FPO**	Data on Thailand annual inflation rate forecast made by the FPO between 2003 and 2022.	20	BOT [27]
**Inf forecast made by NESDC**	Data on Thailand annual inflation rate forecast made by the NESDC between 2003 and 2022.	20	BOT [27]
**Actual Inf**	Actual data on Thailand annual inflation rate between 2003 and 2022.	20	BOT [27]

## Method validation

### Limitations of existing deterministic binary forecast skill verification methods

This study first shows the limitations of existing deterministic binary forecast skill verification methods, namely, PSS, DSS, HSS, ORSS, CSS, and PSI in that they award the maximum value of $A_{i}$ for the perfect forecast of events that are considered easy to forecast which are Alternate and Half-half. They are reported in Table 5.

**Table 5:** The value of $A_{i}$ awarded by PSS, DSS, HSS, ORSS, CSS, and PSI for the perfect forecasts of events considered easy to forecast.

Events	Cell counts in 2 × 2 contingency table				$A_{\mathrm{PSS}}$	$A_{\mathrm{DSS}}$	$A_{\mathrm{HSS}}$	$A_{\mathrm{ORSS}}$	$A_{\mathrm{CSS}}$	$A_{\mathrm{PSI}}$
	*Forecasted/observed outcomes*									
	*Yes/Yes* *(a)*	*Yes/No* *(b)*	*No/Yes* *(c)*	*No/No* *(d)*	[−1, 1]	[−1, 1]	[−1, 1]	[−1, 1]	[−1, 1]	[−1, 1]
**Alternate**	200	0	0	200	1.000	1.000	1.000	1.000	1.000	1.000
**Half-half**	200	0	0	200	1.000	1.000	1.000	1.000	1.000	1.000

The results show that, for the perfect forecasts of Alternate and Half-half, all six deterministic binary forecast skill verification methods, namely, PSS, DSS, HSS, ORSS, CSS, and PSI award the maximum score of 1.000 which can be interpreted as the forecast skill is 100 % better than the random forecast.

### Existing deterministic binary forecast skill verification methods with IF

#### Events considered easy to forecast

To overcome the limitations of PSS, DSS, HSS, ORSS, CSS, and PSI that fail to award the correct scores for forecasting events that are considered easy to forecast as shown in Table 5, IF is introduced. As described in Method details, IF is derived from EEF and FE. Table 6 reports the values of AS awarded to SFM1 and SFM2 for forecasting two types of events which are Alternate and Half-half.

**Table 6:** The values of AS awarded to SFM1 and SFM2 for forecasting events considered easy to forecast.

Events	Cell counts in 2 × 2 contingency table				AS
	*Forecasted/observed outcomes*				
	*Yes/Yes* *(a)*	*Yes/No* *(b)*	*No/Yes* *(c)*	*No/No* *(d)*	[0, 1]
**Alternate**					
- SFM1	1	200	199	0	0.003
- SFM2	199	0	1	200	0.998
**Half-half**					
- SFM1	200	1	0	199	0.998
- SFM2	0	199	200	1	0.003

As shown in Table 6, SFM2 receives the AS of 0.998 for forecasting Alternate while SFM1 receives the AS of 0.998 for forecasting Half-half. The values of AS_SFM1_ and AS_SFM2_ for each type of event are then used to compute the respective values of EEF. Table 7 reports the values of EEF which are equal to 0.995 for both Alternate and Half-half. This indicates that Alternate and Half-half are events that are somewhat easy to forecast correctly since the values of EEF are closer to 1. The next step is to use EEF in combination with FE in order to calculate the value of IF for each type of event. The values of FE, as reported in Table 7, are equal to 1.000 for both Alternate and Half-half, suggesting that the two events occur more frequently. Given the values of EEF and FE, IF can be computed by using Eq. (6). The values of IF for Alternate and Half-half are also reported in Table 7.

**Table 7:** The values of EEF, FE, and IF for Alternate and Half-half.

Events	AS_SFM1_	AS_SFM2_	EEF	FE	IF
	[0, 1]	[0, 1]	[0, 1]	[0, 2]	[0, 1]
**Alternate**	0.003	0.998	0.995	1.000	0.005
**Half-half**	0.998	0.003	0.995	1.000	0.005

Given that Alternate and Half-half are events that are easy to forecast correctly with EEF = 0.995 and occur more frequently with FE = 1.000, the value of IF is equal to 0.005. As described in Method details, the values of IF would be used to multiply $A_{i}$ in order to get $A_{i}^{\text{IF}}$. Table 8 reports the values of $A_{i}^{\text{IF}}$ for the perfect forecasts of events considered easy to forecast which are Alternate and Half-half.

**Table 8:** The value of $A_{i}^{\text{IF}}$ awarded by PSS, DSS, HSS, ORSS, CSS, and PSI each of which is equipped with IF for the perfect forecast of events considered easy to forecast.

Events	Cell counts in 2 × 2 contingency table				$A_{\mathrm{PSS}}^{\mathrm{IF}}$	$A_{\mathrm{DSS}}^{\mathrm{IF}}$	$A_{\mathrm{HSS}}^{\mathrm{IF}}$	$A_{\mathrm{ORSS}}^{\mathrm{IF}}$	$A_{\mathrm{CSS}}^{\mathrm{IF}}$	$A_{\mathrm{PSI}}^{\mathrm{IF}}$
	*Forecasted/observed outcomes*									
	*Yes/Yes* *(a)*	*Yes/No* *(b)*	*No/Yes* *(c)*	*No/No* *(d)*	[−1, 1]	[−1, 1]	[−1, 1]	[−1, 1]	[−1, 1]	[−1, 1]
**Alternate**	200	0	0	200	0.005	0.005	0.005	0.005	0.005	0.005
**Half-half**	200	0	0	200	0.005	0.005	0.005	0.005	0.005	0.005

For the perfect forecasts of Alternate and Half-half, PSS, DSS, HSS, ORSS, CSS, and PSI equipped with IF clearly improve the performance of the original PSS, DSS, HSS, ORSS, CSS, and PSI without IF by awarding a score of 0.005 which is close to the random forecast whose score is set to be 0 as opposed to the original PSS, DSS, HSS, ORSS, CSS, and PSI without IF that award a score of 1.000 for the perfect forecasts of these two types of events. Provided that the reference skill score against which a forecast can be judged is the random forecast whose value is set to be 0 %, these results indicate that the forecasts of Alternate and Half-half, even though they are perfectly accurate, are 0.5 % better than the random forecast when assessed by PSS, DSS, HSS, ORSS, CSS, and PSI equipped with IF in contrast to 100 % better than the random forecast when evaluated by the original PSS, DSS, HSS, ORSS, CSS, and PSI without IF as shown in Table 5.

### Application of IF using Inf forecast as a case study

In order to demonstrate that IF could be used together with the existing deterministic binary forecast skill verification methods in practice, this study employs the data on Inf forecasts made by BOT, FPO, and NESDC between 2003 and 2022 as well as the actual data on Inf during the same period. First, this study reports the results of the evaluation of skills in directional forecasts of Inf made by BOT, FPO, and NESDC that are measured by the existing deterministic binary forecast skill verification methods, namely, PSS, DSS, HSS, ORSS, CSS, and PSI. They are shown in Table 9.

**Table 9:** The values of $A_{i}$ awarded by PSS, DSS, HSS, ORSS, CSS, and PSI for Inf forecasts made by BOT, FPO, and NESDC between 2003 and 2022.

Forecasters	Cell counts in 2 × 2 contingency table				$A_{\mathrm{PSS}}$	$A_{\mathrm{DSS}}$	$A_{\mathrm{HSS}}$	$A_{\mathrm{ORSS}}$	$A_{\mathrm{CSS}}$	$A_{\mathrm{PSI}}$
	*Forecasted/observed outcomes*									
	*Up/Up* *(a)*	*Up/Down* *(b)*	*Down/Up* *(c)*	*Down/Down* *(d)*	[−1, 1]	[−1, 1]	[−1, 1]	[−1, 1]	[−1, 1]	[−1, 1]
**BOT**	10	2	2	6	0.583	0.583	0.583	0.875	0.583	0.577
**FPO**	11	3	1	5	0.542	0.579	0.565	0.897	0.619	0.564
**NESDC**	10	3	2	5	0.458	0.471	0.468	0.786	0.484	0.463

The results show that BOT receives scores ranging between 0.577 and 0.583 except ORSS that gives a score of 0.875 while FPO receives scores ranging between 0.542 and 0.619 except ORSS that awards a score of 0.897. NESDC receives scores ranging between 0.458 and 0.484 except ORSS that awards a score of 0.786. These results indicate that the skill in forecasting the direction-of-change of Inf of BOT is between 57.7 % and 58.3 % better than random forecast based on PSS, DSS, HSS, CSS, and PSI while it is 87.5 % better than random forecast based on ORSS. The skill in directional forecast of Inf of FPO is between 54.2 % and 61.9 % better than random forecast as measured by PSS, DSS, HSS, CSS, and PSI while it is 89.7 % better than random forecast as measured by ORSS. For NESDC, the skill in forecasting the direction-of-change of Inf is between 45.8 % and 48.4 % better than chance according to PSS, DSS, HSS, CSS, and PSI whereas it is 78.6 % better than chance according to ORSS.

Similar to the steps for calculating IF for events considered easy to forecast, the calculation of IF for Inf between 2003 and 2022 begins by calculating the values of AS awarded to SFM1 and SFM2 for Inf forecasts between 2003 and 2022. As reported in Table 10, SFM1 receives the AS of 0.600 while SFM2 receives the AS of 0.400 for Inf forecasts between 2003 and 2022.

**Table 10:** The values of AS awarded to SFM1 and SFM2 for Inf forecasts between 2003 and 2022.

Forecasters	Cell counts in 2 × 2 contingency table				AS
	*Forecasted/observed outcomes*				
	*Up/Up* *(a)*	*Up/Down* *(b)*	*Down/Up* *(c)*	*Down/Down* *(d)*	[0, 1]
SFM1	8	4	4	4	0.600
SFM2	4	4	8	4	0.400

The values of AS_SFM1_ and AS_SFM2_ for Inf forecasts between 2003 and 2022 are then used to calculate EEF which, in turn, would be used together with the value of FE in order to compute the value of IF for Inf between 2003 and 2022. Table 11 reports the values of EEF, FE, and IF for this event.

**Table 11:** The values of EEF, FE, and IF for Inf between 2003 and 2022.

**Inf between 2003 and 2022**	**AS_SFM1_**	**AS_SFM2_**	**EEF**	**FE**	**IF**
	[0, 1]	[0, 1]	[0, 1]	[0, 2]	[0, 1]
	0.600	0.400	0.200	1.200	0.800

As shown in Table 11, the EEF of 0.200 suggests that it is somewhat difficult to accurately forecast the direction-of-change of Inf whereas the FE of 1.200 indicates that, for the direction-of-change of Inf, *Up* occurs slightly more frequently than *Down*. The values of EEF and FE are then used to calculate the value of IF which is equal to 0.800 as reported in Table 11. To calculate the values of $A_{i}^{\text{IF}}$ for BOT, FPO, and NESDC, the value of IF is used to multiply $A_{i}s$ that each organization receives for Inf forecasts between 2003 and 2022 as reported in Table 9. Table 12 reports the values of $A_{i}^{\text{IF}}$ for Inf forecasts made by BOT, FPO, and NESDC between 2003 and 2022. For the purpose of comparison, the values of $A_{i}$ awarded by PSS, DSS, HSS, ORSS, CSS, and PSI without IF for Inf forecasts made by BOT, FPO, and NESDC between 2003 and 2022 as reported in Table 9 are also shown in Table 12.

**Table 12:** The values of $A_{i}^{\text{IF}}$ awarded by PSS, DSS, HSS, ORSS, CSS, and PSI each of which is equipped with IF for Inf forecasts made by BOT, FPO, and NESDC between 2003 and 2022. The values of $A_{i}$ awarded by PSS, DSS, HSS, ORSS, CSS, and PSI without IF for Inf forecasts made by BOT, FPO, and NESDC between 2003 and 2022 as reported in Table 9 are shown in parenthesis.

Forecasters	Cell counts in 2 × 2 contingency table				$A_{\mathrm{PSS}}^{\mathrm{IF}}$	$A_{\mathrm{DSS}}^{\mathrm{IF}}$	$A_{\mathrm{HSS}}^{\mathrm{IF}}$	$A_{\mathrm{ORSS}}^{\mathrm{IF}}$	$A_{\mathrm{CSS}}^{\mathrm{IF}}$	$A_{\mathrm{PSI}}^{\mathrm{IF}}$
	*Forecasted/observed outcomes*									
	*Up/Up* *(a)*	*Up/Down* *(b)*	*Down/Up* *(c)*	*Down/Down* *(d)*	[−1, 1]	[−1, 1]	[−1, 1]	[−1, 1]	[−1, 1]	[−1, 1]
**BOT**	10	2	2	6	0.467 (0.583)	0.467 (0.583)	0.467 (0.583)	0.700 (0.875)	0.467 (0.583)	0.462 (0.577)
**FPO**	11	3	1	5	0.433 (0.542)	0.463 (0.579)	0.452 (0.565)	0.717 (0.897)	0.495 (0.619)	0.451 (0.564)
**NESDC**	10	3	2	5	0.367 (0.458)	0.377 (0.471)	0.374 (0.468)	0.629 (0.786)	0.387 (0.484)	0.370 (0.463)

The results show that PSS, DSS, HSS, CSS, and PSI equipped with IF award BOT scores ranging between 0.462 and 0.467 while ORSS with IF awards a score of 0.700. PSS, DSS, HSS, CSS, and PSI equipped with IF award FPO scores ranging between 0.433 and 0.495 whereas ORSS with IF awards a score of 0.717. NESDC receives scores ranging between 0.367 and 0.387 as assessed by PSS, DSS, HSS, CSS, and PSI equipped with IF while ORSS with IF gives a score of 0.629. These results suggest that the skill in directional forecast of Inf of BOT is between 46.2 % and 46.7 % better than random forecast based on PSS, DSS, HSS, CSS, and PSI equipped with IF while it is 70.0 % better than random forecast based on ORSS with IF. For FPO, its skill in directional forecast of Inf is between 43.3 % and 49.5 % better than chance according to PSS, DSS, HSS, CSS, and PSI equipped with IF while it is 71.7 % better than chance according to ORSS with IF. As measured by PSS, DSS, HSS, CSS, and PSI equipped with IF, the skill of NESDC in directional forecast of Inf is between 36.7 % and 38.7 % better than random forecast while it is 62.9 % better than random forecast as measured by ORSS with IF.

Given that the nature of direction-of-change of Inf cannot be easily forecasted with EEF = 0.200 and its movement is such that *Up* occurs slightly more frequently than *Down* with FE = 1.200, PSS, DSS, HSS, ORSS, CSS, and PSI with IF therefore award scores for BOT, FPO, and NESDC in forecasting the direction-of-change of Inf relatively lower than those awarded by the corresponding deterministic binary forecast skill verification methods without IF by a factor of 0.800. Nevertheless, the overall results indicate that the skills of BOT, FPO, and NESDC in directional forecast of Inf between 2003 and 2022 are better than a fair coin toss.

## Conclusions

This study introduces IF in order to overcome the limitations of the existing deterministic binary forecast skill verification methods, namely, PSS, DSS, HSS, ORSS, CSS, and PSI that award a perfect score for forecasting two broad types of events that are considered easy to forecast which are Alternate and Half-half. By using two hypothetical datasets on events that are considered easy to forecast which are Alternate and Half-half, this study shows that PSS, DSS, HSS, ORSS, CSS, and PSI equipped with IF could improve the performance of original PSS, DSS, HSS, ORSS, CSS, and PSI without IF by awarding the score that are close to the score for no-skill forecast for forecasting these two types of events. In addition, this study employs the forecast and actual data on Inf between 2003 and 2002 in order to demonstrate that IF could be used together with the existing deterministic binary forecast skill verification methods, namely, PSS, DSS, HSS, ORSS, CSS, and PSI to evaluate skills of forecasters, namely, BOT, FPO, and NESDC, in practice. Since deterministic binary forecast skill verification methods are widely used in a number of scientific disciplines as discussed in Background, IF could be used to improve the performance of existing deterministic binary forecast skill verification methods which could benefit forecasters who develop models and/or machine learning algorithms for forecasting binary events. In addition, EEF could be used as a separate tool to preliminarily detect the nature of event whether it is easy or difficult to forecast accurately.

## Limitations

Not applicable.

## Ethics statements

Not applicable.

## CRediT authorship contribution statement

**Thitithep Sitthiyot:** Conceptualization, Methodology, Formal analysis, Writing – original draft, Writing – review & editing. **Kanyarat Holasut:** Methodology, Validation, Writing – review & editing.

## Declaration of competing interest

The authors declare that they have no known competing financial interests or personal relationships that could have appeared to influence the work reported in this paper.

## Data Availability

All data analyzed in this study are available in Supplementary material.

## Appendix Supplementary materials

Image, application 1
